# Label‐Free Molecular Characterization of Protein Aggregates in Differentiated Astrocytes

**DOI:** 10.1002/advs.202515228

**Published:** 2026-01-07

**Authors:** Panagis D. Samolis, Chiara Lazzarini, Rahmetullah Durgun, Barbara Barile, Giorgia Conte, Tamara Posati, Marco Caprini, Grazia Paola Nicchia, Valentina Benfenati, Michelle Y. Sander

**Affiliations:** ^1^ Department of Electrical and Computer Engineering Boston University Boston Massachusetts USA; ^2^ BU Photonics Center and BU Neurophotonics Center Boston Massachusetts USA; ^3^ Consiglio Nazionale delle Ricerche – Institute For Organic Synthesis and Photoreactivity (CNR‐ISOF) Bologna Italy; ^4^ Department of Biosciences, Biotechnology and Environment University of Bari Aldo Moro Bari Italy; ^5^ Department of Pharmacy and Biotechnology University of Bologna Bologna Italy; ^6^ Division of Materials Science and Engineering Boston University Boston Massachusetts USA; ^7^ Department of Biomedical Engineering Boston University Boston Massachusetts USA

**Keywords:** astrocyte processes, astrocytes, mid infrared photothermal microscopy, secondary protein structure, thermal diffusion

## Abstract

Astrocyte cell differentiation to their characteristic starlike morphology with the expression of proteins in microdomains, critical for normal brain function, occurs naturally in‐vivo but can be affected in pathological condition or in cell culture in‐vitro. Analyzing the molecular composition and functional properties of astrocytes in a label‐free manner with sub‐micron spatial resolution can enable detailed insights into their role in brain physio‐pathology. However, simultaneous insights into any structural, molecular, and functional features in unlabelled differentiated astrocytes, without perturbing their natural environment with exogenous tags, has been limited. Using mid‐infrared photothermal imaging, an accumulation of α‐helical signatures for the extended astrocyte processes is observed in differentiated astrocytes on a nanomaterials interface. At the same time, non‐differentiated astrocytes feature a more diverse protein content, rich in *β*‐sheets. Time‐resolved photothermal diffusion measurements indicate a higher interfacial thermal resistance at the astrocyte processes, connecting protein structure with thermal relaxation dynamics experimentally within the same measurement, critical for energy transport and homeostasis. This photothermal multi‐parameter characterization offers unique insights into what chemically and functionally determines healthy astrocytes, paving the way towards a deeper understanding of their differentiation mechanisms. This method allows for the detection of molecular, morphological, and functional signatures associated with pathological state of astrocytes ex‐vivo.

## Graphical abstract

Mid‐infrared photothermal microscopy enables label‐free structural, molecular, and functional imaging of protein aggregates in astrocyte cells. The processes of astrocytes differentiated on a nanomaterial interface are characterized by *α*‐helical signatures combined with enhanced interfacial thermal resistance properties, while the cell soma of non‐differentiated astrocytes feature richer content in *β*‐sheets and faster interfacial heat transfer.

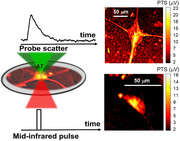

## 1 Introduction

Physiological processes in the brain involve the movement of ions and molecules inside and outside cells within the central nervous system (CNS). The osmotic balance of the extracellular fluid and the maintenance of cell volume are influenced by the generation of transient osmotic micro‐gradients of ions and molecules, generated by neural cell activity [1]. Astrocytes, a type of glial cell, play a crucial role in regulating brain homeostasis through membrane proteins forming ion and water channels, which are assembled in micro‐domains expressed in the endfeet of astrocytes. Aquaporin‐4 (AQP4), calcium channels belonging to Transient Receptor Potential Vanilloid 4 Superfamily (TRPV4), and chloride channels called Volume‐Regulated Anion Channels (VRAC) are key membrane protein channels involved in astrocytic brain volume homeostasis [2]. AQP4 is selectively expressed in astrocytic endfeet that face blood vessels and primarily facilitates faster, hydrostatically and osmotically driven water transport in the brain [3].

Alterations in the structural and functional properties of these membrane proteins can disrupt cellular function and contribute to the development of various chronic diseases, such as Alzheimer's, multiple sclerosis, epilepsy, glioma, and ischemia. These disruptions occur through the accumulation of protein aggregates, dysfunction of cellular membranes, and abnormal immune responses [4, 5, 6, 7].

A major issue in studying astrocytic mechanisms beyond their function and associated changes, in vitro, is that the above‐mentioned membrane protein specialized microdomains in classical cell culture model systems do not resemble those observed in vivo. In our previous study, we demonstrated that rat primary astrocytes grown on nanostructured interfaces based on hydrotalcite‐like compounds (HTlc) in vitro are differentiated and display molecular and functional properties of astrocytes in vivo, such as the functional expression of inwardly rectifying K^+^ channel (Kir 4.1) and AQP4, TRPV4 and VRAC at the astrocytic elongations, as well as and improved capability in water diffusion, cell volume regulation and potassium buffering [8, 9]. Nonetheless, we showed that the same in vitro model, which displayed actin microdomains at the astrocyte's boundaries, responds more efficiently to homeostatic challenges, such as changes in extracellular osmolarity or in potassium concentration [10]. However, information is lacking to correlate the structure and the functional properties of these proteins.

Current tools for correlating the structure and function of these proteins include X‐ray crystallography, transmission electron microscopy (TEM), and Nuclear Magnetic Resonance (NMR) spectroscopy. While these methods are invaluable for structural determination, they do not provide a complete picture of the relationship between protein structure and function. The integration of additional methodologies is essential for advancing our understanding of these complex biological macromolecules [11]. Limited in vitro imaging has been performed on glia cells for molecular composition analysis and tracking of morphological changes. Super‐resolution fluorescence microscopy by stimulated emission depletion (STED) revealed stronger actin dynamics in the boundary region of non‐differentiated cells [10]. In a label‐free approach with optical diffraction tomography, in vitro astrocyte studies over 7 days allowed capturing their transition to flattened, larger area shapes [12]. Thus, label‐free imaging methodologies that can resolve molecular secondary structure can significantly advance our insight into biological morphology.

Mid‐infrared (mid‐IR) photothermal imaging provides a powerful label‐free modality that can extract rich chemical and structural information present in the molecular fingerprint region (3–12 µm) with submicron spatial resolution [13, 14, 15, 16, 17, 18, 19, 20, 21, 22, 23, 24, 25]. This has enabled the study of protein secondary structure in biological specimens since the peak of mid‐IR vibrational resonances depends on the backbone conformation, e.g. as is the case for the Amide I band [26]. Hence, *β*‐sheet protein clusters in the perinuclear region of fibroblast cells [27] were identified, *β*‐sheets in tau fibrils [18] were visualized in three‐dimensions and amyloid‐*β*‐proteins were detected in neurons [28]. With live‐cell imaging, cellular dynamics associated with brain cells were monitored in real‐time, e.g. the cell division processes of oligodendrocytes [29], while in neurons, intercellular protein‐rich vesicle transport was visualized along the axon [29].

However, while structural information has been previously localized with this technique, no direct correlation with the functional properties of such cell domains, including thermal diffusion dynamics has been demonstrated. With time‐resolved photothermal microscopy, one of the most recent advancements in this method, complementary information to absorption properties is provided by measuring the heating and diffusion transient dynamics of the photothermal signal. This has found applications in resonant background signal subtraction [14, 22, 30] and spatial mapping of thermal diffusion in neurological axon bundles [14], brain cancer cells [31], as well as polymer nanoparticles [22, 30]. While currently mid‐IR photothermal imaging of glia cells has been fairly selective [32], the capabilities of imaging cell differentiation in vitro with sub‐micron spatial resolution in real‐time can offer new insights into brain communication, memory formation, or aging.

In this work, we use time‐resolved and multi‐spectral photothermal imaging to simultaneously extract thermal diffusion as well as molecular properties of micro‐domains in astrocytes. To this end, we perform the analyses on primary rat neocortical astrocytes grown on traditional poly‐D‐lysine (PDL) substrates, which typically retain an undifferentiated and polygonal shape, and on astrocytes grown on HTlc, which provides an effective and biocompatible platform for driving astrocytic molecular and functional differentiation in vitro.

We demonstrate that a diverse pool of protein secondary structure, rich in *β*‐sheets, characterizes non‐differentiated cells, while the processes of differentiated cells are dominated by *α*‐helical protein signatures. This change is accompanied by increased levels of interfacial thermal resistance in the processes of differentiated cells compared to the cytoplasm of non‐differentiated cells. This study advances insights into how structural changes of astrocytic microdomains are linked to their functional roles, including the regulation of ion, water balance, and thermal diffusion dynamics in vitro, that can be compared to those occurring in astrocytes in vivo. The presented molecular, structural, and functional differences between polygonal and differentiated cells serve as a blueprint for astrocyte cell imaging in healthy and pathological states.

## 2 Results

### 2.1 Multi‐Modular Mid‐Infrared Photothermal Microscope

**FIGURE 1:**
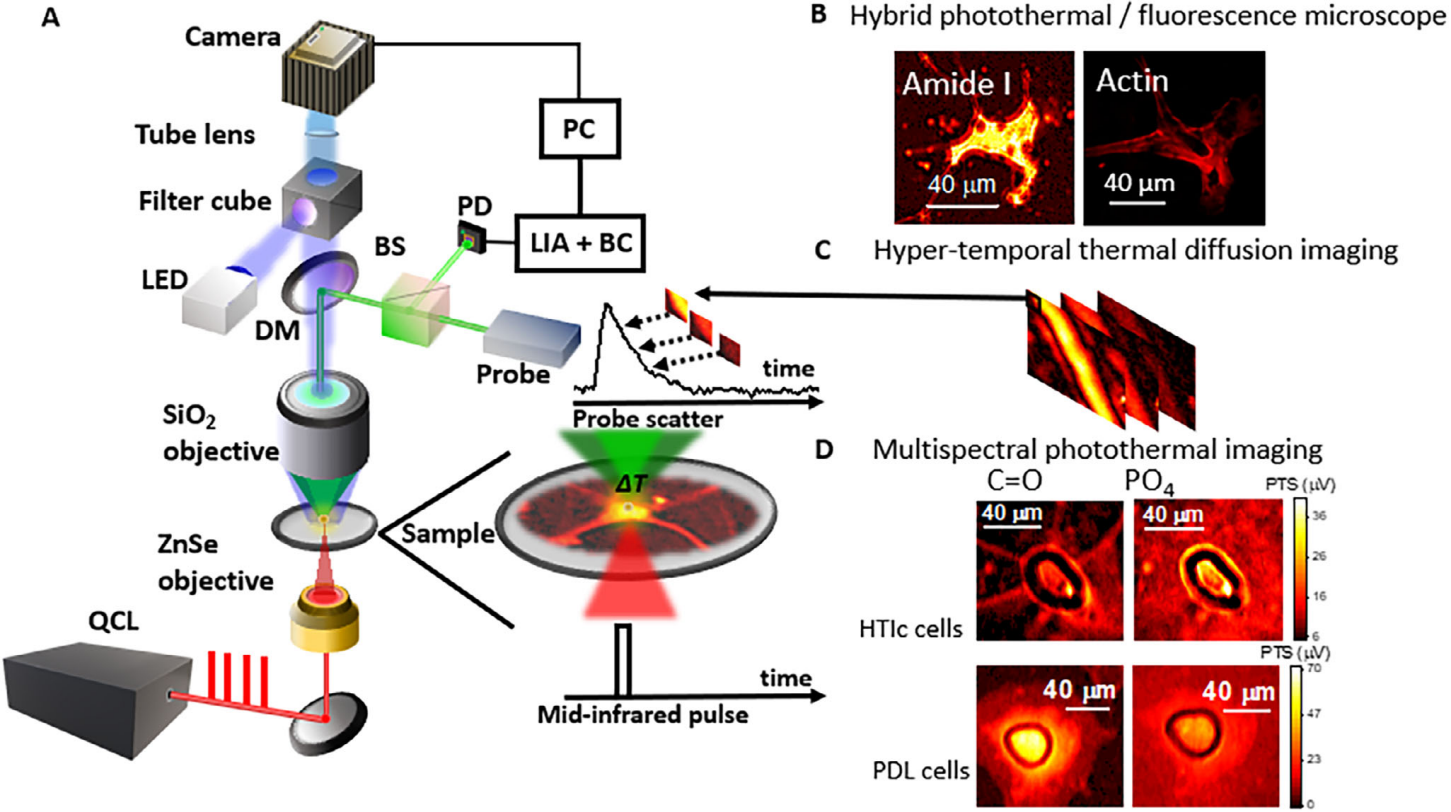
Multi‐modular mid‐infrared photothermal microscope for biomedical imaging. (A) Experimental setup (LIA: lock‐in amplifier, DM: dichroic mirror, BS: beam splitter, PD: photodiode).(B) Cross‐registration of wide‐field fluorescence microscopy with photothermal mid‐IR imaging. (C) Hypertemporal image stack that maps thermal diffusion properties. (D) Multi‐spectral imaging reveals different structural and chemical information when targeting protein resonances (λ = 1660 cm^−1^) versus nucleic acid resonances (λ = 1080 cm^−1^) in differentiated astrocyte cells grown on HTlc as well as non‐differentiated astrocyte cells grown on PDL.

The photothermal microscope system is based on VIPPS (Vibrational Infrared Photothermal and Phase Signal) imaging [27], shown in Figure 1A, and consists of a shorter wavelength probe laser combined with a pulsed quantum cascade laser (QCL) as a pump, with a tunable emission in the mid‐IR region that targets characteristic vibrational resonances. Using thermal lensing, sub‐diffraction‐limited chemical imaging of mid‐IR signatures with a spatial resolution around 750 nm can be achieved (see Methods). A direct cross‐registration of fluorescence and photothermal amplitude signal (PTS) images can be performed (see Figure 1B). Time‐resolved photothermal images are obtained with a boxcar detection system in a single measurement so that in hyper‐temporal image stacks the heating and thermal diffusion dynamics can be visualized, as shown in Figure 1C (see Methods).

Multi‐spectral amplitude and thermal diffusion imaging is performed by tuning the emission wavelength of the pump. To gain insight into structural and functional differences between undifferentiated and differentiated astrocytes, we analyze and compare primary cortical astrocytes grown on standard glass coverslips coated with PDL or with a drop‐cast film of HTlc nanoparticles [8]. Astrocyte processes setting allowed the comparison of structure and function between undifferentiated astrocytes (here reported as PDL cells) and differentiated ones (referred to as HTlc cells in this paper). When grown on an HTlc substrate, PTS cell imaging reveals unique chemical, structural, and functional signatures in domains of differentiated cells that are not found in PDL cells. In Figure 1D, the multispectral images of an astrocyte HTlc cell and a PDL cell are presented. Analyzing the proteins within the Amide I band (for a pump wavenumber λ targeting the C═O bonds in carboxyl groups at 1660 cm^−1^), the structural shape of the cell cytoplasm reveals the star‐like extensions characteristic of differentiated astrocytes for the HTlc cell. For the PDL cell, a circularly shaped cytoplasm is resolved. Alternatively, targeting PO_4_ bonds in phosphate groups that are present in nucleic acids (for a pump wavenumber λ at 1080 cm^−1^), a high signal associated with nucleic acids concentrated in the cell nucleus region are visible against a substrate residual background due to phosphate buffer solution from the sample preparation. Hence, our hybrid photothermal microscope system can map chemical content, as well as thermal diffusion properties, combined with fluorescence microscopy for cross‐registration purposes.

### 2.2 Multi‐Spectral Photothermal Imaging of Differentiated Astrocyte Cells Grown on a HTlc Substrate Reveals α—Helical Composition of Their Processes

**FIGURE 2:**
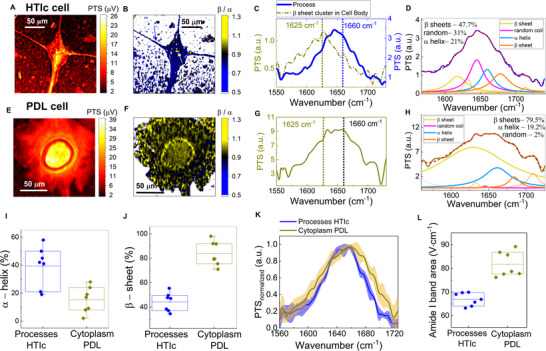
Secondary protein structure in astrocytes. (A) PTS image of differentiated HTlc cell at the Amide I protein band (λ = 1660 cm^−1^). (B) Ratio image *β*‐sheet over *α*‐helix proteins, highlighting a low *β/α* ∼ 0.78 in the HTlc processes while the cell body contains regions with *β/α*∼ 1.5. (C) Characteristic spectrum at the HTlc cell processes (solid blue) and at clusters of *β*‐sheets (dashed dotted dark yellow). (D) Deconvolved fitted spectra of HTlc cell process (dashed red), composed of 21% of *α*‐helix content (cyan), 47% of *β*‐sheet (yellow for low wavenumber *β*‐sheet, orange for high wavenumber *β*‐sheet), and 31% of random coil (magenta) content. (E) PTS image of non‐differentiated pdl cell at the Amide I protein band. (F) Ratio imaging *β/α* indicates a more homogenous distribution with *β/α* ∼ 0.9. (G) Characteristic broad Amide I spectra at cell body (dark yellow). (H) Deconvolved fitted spectra of PDL cell cytoplasm (dashed red), demonstrates twice the amount of *β*‐sheet content at 79% (yellow for low wavenumber *β*‐sheet, orange for high wavenumber *β*‐sheet). Box chart graphs of *α*‐helix content (I) and *β*‐sheet content (J) in HTlc cell processes and PDL cell cytoplasm. (K) Normalized mean Amide I band spectra from the HTlc cell processes (solid blue) and from cytoplasm of non‐differentiated cells (solid yellow), with the latter being broader and with higher content in β‐sheets. (L) Amide I band spectral area (from 1600 to 1730 cm^−1^) measured at the HTlc cell processes (blue circles) and cytoplasm of PDL cells (yellow triangles).

The PTS image at the Amide I band peak (1660 cm^−1^) of a differentiated HTlc cell (from a primary rat astrocyte) is shown in Figure 2A with processes extending from the main cell body. By tuning the pump emission, we investigate the relative signal contributions at wavenumbers associated with different secondary protein conformations present in the Amide I band, specifically beta (*β*) sheets (at λ = 1625 cm^−1^ or λ = 1675 cm^−1^) as well as alpha (*α*) helical proteins (λ = 1660 cm^−1^). In Figure 2B, the ratio of the image obtained at 1625 cm^−1^ over 1660 cm^−1^ (*β*/*α* images) is shown (see Methods), with the false color bar upper and lower limits set to the range of β/α values present in the cell region. The astrocyte processes grown on HTlc contain lower β/α ratio values <0.9, while localized domains exist in the cell body with β*/α* values surpassing 1. Representative spectra from the HTlc cell (see Methods) are shown in Figure 2C, collected at the processes (solid blue‐ see arrow in Figure 2B) with a peak at 1640 cm^−1^ and β/α value at 0.77. Spectra collected at the domains of higher β/α values, see Figure 2C (dashed dotted yellow – see arrow in Figure 2B) present a clear red shift of the Amide I peak to 1626 cm^−1^, resulting in β/α ratio value of 1.5. Individual secondary protein contributions are identified with a second derivative analysis combined with spectral deconvolution (see Methods), shown in Figure 2D for the spectra collected at the astrocyte processes. The relative percentages of *β*‐sheet (see yellow for low wavenumber *β*‐sheet, see orange for high wavenumber *β*‐sheet) and *α*‐helix (shown in cyan) contributions are found to be 47% and 21%, respectively, with an additional 31% contributions from random coil (shown in magenta). Similar analysis for additional HTlc cells is presented (see Figure S1).

In contrast, cells grown on a PDL substrate rather feature a polygonal shape. The PTS image at the Amide I band peak (1660 cm^−1^) of a PDL cell is shown in Figure 2E. The β/α ratio, shown in Figure 2F, is characterized by a more spatially homogeneous distribution with values close to and even exceeding 0.9. The Amide I spectra collected at the cell body, cf. Figure 2G, is fairly broadband with high peaks at wavenumbers associated with α‐helical and *β*‐sheet structures and *β/α* ≈ 0.9. Likewise, the deconvolution analysis shown in Figure 2H, reveals almost twice the contributions from *β*‐sheets for non‐differentiated cells, up to 79% (see yellow for low wavenumber *β*‐sheet, see orange for high wavenumber *β*‐sheet). Similar analysis for additional PDL cells is presented (see Figure S2). Overall, the mean *β/α* value was determined to be around 0.96 ± 0.07 (number of cells N = 6, associated area for evaluation A = 25 949 µm^2^) for the cell body of PDL cells compared to 0.78 ± 0.09 (N = 6, A = 11,606 µm^2^) for the processes of HTlc cells (see Figure S3). This increase was found to be statistically significant (*p*‐value at 0.0003). In addition, the spectral deconvolution reveals a mean value of α‐helical content at the HTlc cell processes of around 40% (N = 6), which is 2.6‐fold larger than the content found in the cytoplasm of the PDL cells with a mean value of 15% (N = 6) (see Figure 2I ‐ with box boundaries corresponding to values one standard deviation away from mean value denoted by a line). Similarly, a 1.8 ‐fold increase in β‐sheet content is reported with the mean increasing from 45% for HTlc cell processes up to 85% for the PDL cell cytoplasm (see Figure 2J‐ with box boundaries corresponding to values one standard deviation away from the mean value denoted by a line). It is also worth noting that a random coil secondary protein structure was found uniquely in selective HTlc cell processes, but which consistently had less than 3% contributions in the PDL cell cytoplasm (see Figure S4).

This increase in β‐sheet content results in a broader Amide I spectral band. In Figure 2K, the mean normalized spectra from the cytoplasm of 7 PDL cells (see Figure 2G, solid dark yellow) and 7 HTlc cell processes (N = 5) (see Figure 2C – solid blue) are shown, with the corresponding error margins in the shadowed area. The high contributions of non‐α‐helical protein conformations, consisting mostly of β‐sheets, result in the broadening of the Amide I spectral peak. The area underneath the Amide I spectrum (from 1600 to 1725 cm^−1^) for each cell is shown in the box chart graph in Figure 2L, with box boundaries corresponding to values one standard deviation away from the mean value denoted by a line. The mean spectral area from the cytoplasm of PDL cells at 81.7 ± 5.3 V · cm^−1^ (N = 7) is found to be ∼ 1.3‐fold larger than the corresponding mean spectral area from the HTlc cells processes at 66.9 ± 2.7 V ·cm^−1^ (N = 5).

### 2.3 Boxcar Time Resolved Photothermal Imaging Reveals Slower Thermal Diffusion Times at the Interface of Cell Processes in Differentiated Astrocytes Compared to Cytoplasm of Non‐Differentiated Cells

**FIGURE 3:**
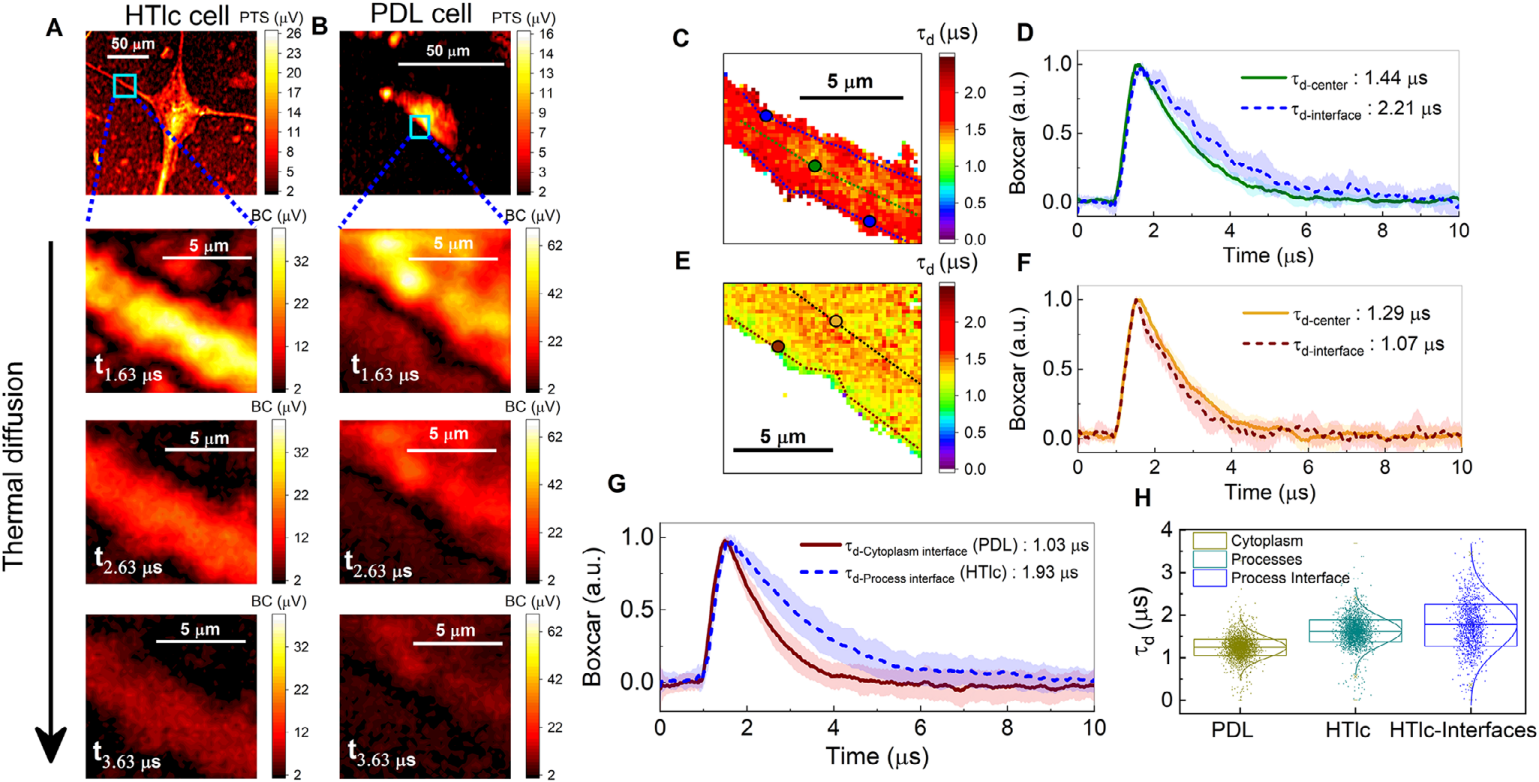
Thermal diffusion dynamics characterizing astrocyte cell processes. Boxcar photothermal images of (A) the cell process from a differentiated astrocyte and (B) the cytoplasm interface from a non‐differentiated astrocyte cell during the diffusion window (1 µs time points). (C) Time decay constant (τ_d_) image of the HTlc cell process, with the interfaces showing larger τ_d_ values than the cell process center. (D) Mean time curves from process interface and center regions denoted by dashed lines across blue and green dots respectively, with τ_d‐interface_ = 2.21 µs > τ_d‐center_ = 1.44 µs. (E) Time decay constant image of the PDL cell cytoplasm, with high signal cytoplasm areas having slightly longer *τ* values than the cytoplasm interface. (F) mean time curves from cytoplasm interface and center region denoted by dashed lines across brown and yellow dots respectively, with τ_d‐interface_ = 1.07 µs < τ_d‐center_ = 1.29 µs. (G). Normalized mean transient curves at the interface of the HTlc cell processes (N = 4 – solid blue) and of the PDL cell cytoplasm (N = 4 – solid brown) with τ_d‐HTlc ‐interface_ = 1.93 µs > τ_d‐PDL‐interface_ = 1.03 µs. (H) Box chart graph of *τ* values measured at the cytoplasm of PDL cells and at the HTlc cell processes with a mean τ at the cytoplasm of PDL cells of τ_d‐PDL_ = 1.2 ± 0.2 µs (N = 4, A = 210 µm^2^), τ_d‐HTlc_ = 1.6 ± 0.3 µs (N = 4, A = 284 µm^2^) and τ_d‐HTlc‐interface_ = 1.8 ± 0.5 µs (N = 4, A = 48 µm^2^) at the HTlc processes.

Aside from molecular morphology, the thermal diffusion dynamics of the HTlc cell process interfaces can be characterized with our photothermal imaging system with boxcar capabilities (see Methods). Representative images (pump wavenumber of 1660 cm^−1^) during the diffusion window, are shown in Figure 3A,B for an HTlc cell process interface of an HTlc cell and for the cytoplasm interface of a PDL cell, respectively. The 2D mapping of the 1/e time decay constant τ, characterizing the rate of thermal diffusion (cf. Figure 3C, see Methods) indicates a spatial heterogeneity, in particular across the HTlc cell process interface, where larger time decay constants are observed (see blue dashed lines in HTlc cell process). The mean transient curve from 10 points selected across the high signal region of the HTlc cell process center (see dashed line across the green dot) is plotted in Figure 3D alongside the mean transient curve from 10 equally distant points collected from both sides of the HTlc cell process interface (0.5 µm offset from process edge – see dashed line across the blue dots). A clear difference in the time decay constant is highlighted with a 1.5‐fold slower decay at the process interface, as shown in Figure 3D. Overall, for the 5 cell processes studied from 4 HTlc cells, the decay constants for the signal collected at the cell process center are generally smaller compared to the cell process interface (see Figure S5), indicating an increased thermal resistance at the cell process interface. For the transient dynamics at the cytoplasm interface of PDL cells, a homogeneous distribution of time decay constant τ is shown in the equivalent time decay images (cf. Figure 3E, see Methods). Similarly, the mean transient curve from 10 points selected at high signal areas of the cytoplasm cell body (see dashed line across the yellow dot) as well as the mean transient curve from 10 points across the cytoplasm interface (see dashed line across the brown dot), are shown in Figure 3F. The interface versus cell body dynamics shows similar behavior, indicating the absence of higher interfacial thermal resistance effects. The latter behavior is also presented for three additional PDL cells (see Figure S6).

Investigating the diffusion properties sheds a new methodology for characterizing differentiated and non‐differentiated astrocytes. The mean time trace from 50 points collected from 5 HTlc cell process interfaces of 4 different HTlc cells (N = 4) as well as from cytoplasm interfaces of 4 different PDL cells (N = 4) are shown in Figure 3G, with the corresponding error margins in the shadowed regions. A 1.8‐fold slower decay constant is confirmed for the HTlc cell process interfaces with τ_d‐HTlc‐interface_ = 1.93 µs > τ_d‐PDL‐interface_ = 1.03 µs.

The overall τ values from the PDL cell cytoplasm, including interface and center regions, fall within a range of τ_d‐PDL_ = 1.2 ± 0.2 µs (N = 4, A = 210 µm^2^), as shown in the box chart graph in Figure 3H (dark yellow dots). In Figure 3H, the box boundaries correspond to the 16% and 84% percentile values, containing the data points within one standard deviation of the mean. The time decay constants for the HTlc cell processes (c.f. Figure 3C; Figure S5 – see Methods) are larger at the interface compared to the process center regions. Thus, the time decay distribution is broader and more inhomogeneous for the HTlc cell process, as demonstrated in Figure 3C,D, with a 1.7‐fold larger standard deviation and an overall 1.3‐fold larger mean at 1.6 ± 0.3 µs (N = 4, A = 284 µm^2^) (cyan dots) (see Figure 3H). The distribution of τ along the astrocyte interfaces (distanced 0.5 µm from the edge) is found to have a mean τ_d‐HTIc‐interface_ = 1.8 ± 0.5 µs (N = 4, A = 48 µm^2^) being 1.4‐fold larger than the mean τ associated with the cytoplasm of PDL cells. More than 50% of the data from process interfaces falls in a range that is larger than one standard deviation away from the mean time decay constant found in the data set for PDL cells.

## 3 Discussion

In this study, chemical, structural, and functional properties of astrocyte processes in differentiated cells are determined with time‐resolved mid‐IR multispectral photothermal microscopy. First, differences in secondary protein structure between astrocytic processes, and the soma of non‐differentiated astrocytes can be linked to the presence of membrane protein aggregates specifically found in microdomains formed at astrocyte endfeet. Such as AQP4, the main mammalian water channel membrane protein found in astrocyte endfeet. Variations in intracellular water content can directly affect the concentration of ions and signaling molecules within cells, leading to changes in cellular activity and communication between cells [33]. To maintain homeostasis, cells employ mechanisms such as Regulatory Volume Decrease (RVD) and Regulatory Volume Increase (RVI), including the movement of ions into and out of cells, which helps restore a stable state [9]. Aquaporin proteins play a critical role in cell volume regulation, neural excitation, signal transmission, synaptic plasticity, and are involved in neurogenesis, cell migration, and energy metabolism within the brain [34]. Each AQP4 monomer consists of six membrane‐spanning α‐helical domains, essentially a barrel surrounded by six *α*‐helixes (as determined by atomic force microscopy [35]), with very little *β*‐sheet presence [36]. AQP4 in astrocytes is physiologically expressed as aggregates of tetramers, known as Orthogonal Arrays of Particles (OAPs) [37]. The anchoring of these OAPs at the astrocyte's endfeet depends on the direct interaction of the AQP4 C terminus and α‐syntrophin, which connects AQP4 to the dystrophin‐associated protein complex and the underlying cytoskeleton [38, 39]. This polarized localization is essential for AQP4 to effectively support astrocytic functionality.

Another important membrane protein is TRPV4, a polymodal sensor activated by thermal stress, cell volume changes, and anisotonic challenges across the plasma membrane [40]. In the cortex, TRPV4 is predominantly expressed on astrocytic membranes near blood vessels and mediates osmotically induced calcium signaling [41]. The efflux of chloride (Cl^−^) and organic osmolytes through VRAC is essential for restoring physiological cell volume during the RVD process [40]. The transmembrane region of TRPV4 mainly consists of six helices with significantly less β‐sheet presence [42]. Considering the importance of astrocyte microdomains in transmembrane regions in vivo, methods to study the structure and function of these clusters are foreseen.

Previous studies indicated that seeding astrocytes on HTlc nanostructured films promotes the morphological differentiation in vitro of astrocytes, which was accompanied by upregulation in the expression and function of AQP4, Kir4.1, of calcium signaling by TRPV4, and of osmosis‐induced chloride currents by VRAC [43]. Notably, the ability of the cell to respond to osmotic challenges through RVD and RVI was also improved. However, while these functional outcomes were observed, little insight into the structural and conformational protein modifications induced by differentiation has been available so far.

With the present work, we closed this gap by utilizing the sensitivity of the Amide I band to backbone protein conformation. It was found that astrocytic processes in differentiated cells have a stronger accumulation of *α*‐helical proteins, with β/α ratio values around 0.78 ± 0.09, and with spectral deconvolution revealing mean values for *α*‐helix and *β*‐sheet content around 41% and 45%, respectively. Localized clusters of β‐sheets are also found in the main cell body interior (with *β/α* ratio values from 0.9 up to 1.5). Non‐differentiated cells have a larger diversity in protein conformation, including high content in *β*‐sheets in the boundary as well as the bulk of the cell, with *β/α* ratios around 0.96 ± 0.07. Spectral deconvolution reveals additionally for non‐differentiated cells close to 5.6‐fold larger *β*‐sheet content (∼85%) and a 2.7‐fold lower mean α‐helix content (15%) when compared to astrocyte processes of differentiated cells. This was also confirmed by their larger overall area of the Amide I spectral band compared to astrocytic processes (88 V · cm^−1^ >70 V · cm^−1^). These results support the tenet of the presence of α‐helical protein aggregates in microdomains of astrocytic processes in vitro.

The coexistence of α‐helical and random‐coil conformations observed exclusively in differentiated astrocytes suggests a differentiation‐dependent increase in structural heterogeneity. This may reflect the upregulation of membrane channels (including AQP4 isoforms), cytoskeletal reorganizers, and intrinsically disordered regulatory proteins. This enrichment of random coil regions in regions rich with *α*‐helical content reflects likely a class of hybrid scaffold or membrane‐cytoskeletal linker proteins, combining structural rigidity with flexible elements that allow dynamic remodeling in astrocytic processes, with α‐syntrophin as one example to recruit other proteins [3]. In addition, predominantly *α*‐helical transmembrane proteins, including AQP4 [44] and GFAP (glial fibrillary acidic protein) [45] contain disordered regions as well. In contrast, the absence of this signature in PDL cells likely reflects a more compact and stable proteome with fewer partially folded or disordered domains.

The broader Amide I signatures observed in non‐differentiated astrocyte cell bodies could be related to proteins with a non‐*α*‐helical secondary structure, including growth factors like TGF‐*β*, which regulates glia cell differentiation. F‐actin is another prominent protein composing the cell cytoskeleton, consisting of both *α*‐helical and *β*‐sheet structures in its subdomains [46]. However, the percentage of *α*‐helical to *β*‐sheet domains varies from individual filaments to more bundled and crosslinked filaments. 2D‐IR spectroscopy studies found that strongly bounded F‐actin are characterized by a decreased content in β‐sheets [47], while mid‐IR photoinduced force microscopy studies have demonstrated broader Amide I signatures for individual non‐crosslinked filaments [48]. F‐actin present in differentiated astrocyte processes of rat and human cells is an example of bundled actin stress fibers [10, 49] with high homogeneity in their orientation, while F‐actin present in non‐differentiated PDL cells is characterized by a higher diversity in orientation [10]. It should be noted that the observed structural differences between the differentiated and non‐differentiated cells are likely influenced by a localized reorganization of membrane microdomains and hence do not necessarily reflect a global shift from α‐helical to β‐sheet structures in the protein content of the whole proteome [50]. Thus, the combination of membrane proteins (that consist up to 25–30% of the total proteins [51, 52]) together with an additional localized and partial structural rearrangement of membrane‐associated proteins into microdomains [53] can result in the observed spectroscopic shifts.

Differences in F‐actin structure at cell boundaries naturally result in differences in functional properties, including stronger actin dynamics and higher mechanical plasticity found in PDL cytoplasm interfaces compared to the stiffer processes of HTlc cells [10]. This was also supported by the lower proliferation rate found in HTlc cells [8]. When exposed to a temperature gradient, actin filaments demonstrate a unidirectional sliding across the filament orientation as a pathway for heat transfer [54]. Thus, the higher diversity of filament orientation in PDL interfaces can provide more pathways for heat transfer and could explain the faster diffusion times observed compared to HTlc cell processes. In addition, protein secondary structure can influence heat transfer dynamics [55], with molecular simulations highlighting differences in vibrational lifetime [56] and higher thermal diffusivity values for beta barrels attributed to larger mean free path during vibrational energy transfer [57]. The *α*‐helical membrane proteins, like the ones found in HTlc cell processes, are also more hydrophobic in nature compared to beta barrel proteins [58], a property that can reduce the thermal conductance compared to more hydrophilic interfaces [59].

All the above suggest an increased interfacial thermal resistance in HTlc cell processes compared to the cytoplasm of PDL cells, which is demonstrated by the overall 2.5‐fold slower decay constant.

The latter data might be in line with the improved ability of astrocytes on HTlc to permeate water, potassium, and chloride [8, 9], and to react and balance anisotonic challenges [9] or to rise in potassium concentrations [10].

We could in fact hypothesize that *α*‐helical–rich membrane proteins, such as AQP4 and TRPV4, and their aggregation into highly ordered micro‐domains might occur via the dystrophin‐associated protein complex, which are directly relevant to homeostatic but also to metabolic function of astrocytes [3, 44]. In fact, the polarized localization of AQP4, Kir4.1, and TRPV4 in perivascular endfeet through syntrophyns in vivo supports water and ion homeostasis, which is essential for neuronal metabolic support. On the other hand, the absence of perivascular syntrophyns alters potassium and water permeability of astrocytes in vivo, leading to slower formation of brain edema in pathological conditions as hyponatremia [60]. Similarly, the structural organization of membrane protein complexes in differentiated astrocytes may reflect their readiness and capability to fulfill metabolic, signaling, and supportive roles in the brain, during neural development [61, 62].

## 4 Conclusions

Overall, we have demonstrated the unique capabilities of time‐resolved mid‐IR multispectral photothermal microscopy to combine chemical protein structure identification with characterization of thermal dynamics. The application of this technology to astrocyte cell imaging revealed the dominant protein secondary structure in their processes and their associated thermal diffusion properties. This label‐free imaging tool stands as a novel approach for studying astrocyte differentiation mechanisms and the role of astrocytic microdomains protein assembly in healthy or pathological states, such as glioma or epilepsy, where the structure and the function of astrocyte microdomains are known to be compromised. Astrocytes are very plastic cells, and their morphology changes in different stages of neural development, or as a cause of injury, as well as in response to synaptic activity [63]. By linking structural rearrangements at the level of protein complexes with the known functional capacities of astrocytes, our approach provides a bridge between molecular architecture and physiological relevance, suggesting that the differentiation‐associated reorganization of the proteome likely contributes to and accompanies the acquisition of multiple astrocytic functionalities.

Immature astrocytes in the developing CNS support neuronal maturation and possess staminal like properties. Mature astrocytes partially lose these functions but gain new functionalities that are essential for adult CNS homeostasis. The latter function is not restricted to ions and water homeostasis but also to the metabolic support of neurons [63]. In pathological conditions, astrocytes become “reactive,” which disrupts their mature homeostatic functions and reactivates some immature astrocyte‐like properties, suggesting a partial reversal of astrocyte maturation [63, 64, 65].

In this respect, future in‐depth analysis of the difference between the bulk and the boundaries of astrocytes might reveal structural/functional correlations related to the well‐known role of astrocytes for trophic and metabolic support of brain function. Nonetheless, given the heterogenicity of astrocytes belonging to different brain regions, we expect that the results reported here underline the feasibility of our approach to provide information on fingerprints and signatures to classify and clarify the diversity of these cells and their role in neural development, neuro‐pathological condition or in the cross‐talk between astrocytes and nanomaterial interfaces.

## 5 Experimental Section/Methods

### 5.1 Cell Culture Preparation

Primary cortical astroglial cultures from rats were established. Briefly, after the meninges were removed, the brains of 1‐2‐day‐old Sprague Dawley pups (P0–P2) were mechanically dissociated and transferred to cell culture flasks containing DMEM‐GlutaMAX medium supplemented with 15% fetal bovine serum, 100 U/ml penicillin, and 100 mg/ml streptomycin (all sourced from Gibco‐Invitrogen). The flasks were kept in a humidified incubator at 37 °C with 5% CO2 for 3 to 4 weeks, with medium changes every 3 days. Before each medium change, the flasks were gently shaken to detach microglial cells from the astrocytic monolayer. Once the cultures reached confluence, the astroglial cells were enzymatically dissociated using trypsin‐EDTA. The cells were then seeded at a high density on HTlc substrates [15000 cells] and maintained in culture medium containing 10% fetal bovine serum. After 5 days in vitro (DIV) following re‐plating, the samples were fixed with 4% paraformaldehyde, washed three times with DPBS (Dulbecco's Phosphate‐Buffered Saline, Gibco), and then mounted using 6 µl of DPBS. Protocols followed Italian animal protection regulations, with approval from the bioethics committees at the University of Bologna and the Ministry of Health (ID 1138, code number 2DBFE.N.3CN, previous protocol number 360/2017‐PR), under the supervision of the veterinary commission responsible for animal welfare at the University of Bologna. Efforts were made to minimize both the number of animals used and their suffering.

### 5.2 Synthesis of HTLc Nanoparticles

A colloidal aqueous dispersion of ZnAl‐HTlc nanoparticles with the formula [Zn_0.72_Al_0.28_(OH)_2_] Br_0.28_ 0.69 H_2_O was prepared using the double‐micro emulsion method. The chemical composition of the ZnAl‐HTlc was determined through TG/DTA and ICP‐OES analysis. For film preparation, 200 µL of the ZnAl‐HTlc colloidal dispersion at a concentration of 1 mg/mL was dropped onto a 25.4 mm diameter CaF_2_ substrate surface in order to achieve a final concentration of 0.2 mg/mL for each substrate. Then the HTlc were dried for 24 h in a sterile hood.

### 5.3 Multi‐Modular Mid‐Infrared Photothermal Setup

The pump source (QCL) has a broad tunable emission in the mid‐infrared from 1360 to 1800 cm^−1^, from 2665 to 2995 cm^−1^ and from 900 to 1200 cm^−1^. The QCL output is pulsed at a repetition rate of 100 kHz and a pulse duration of 500 ns. It is focused via a 0.25 numerical aperture (NA) infrared transparent ZnSe objective with an estimated pump focal spot size at 12 µm. The total power incident on the sample amounts to 2 mW for the protein absorption at the Amide I peak spectral band at a wavenumber of 1660 cm^−1^. The 980 nm laser diode fills the back aperture of a 0.65 NA glass objective, resulting in a probe diffraction‐limited focal spot size of 750 nm. The interaction of the focused probe beam with the pump‐induced thermal lens results in a modulated backscattered probe field, which is collected via a 50:50 beam splitter (BS) and focused via a 15 mm focal length lens on a silicon photodiode (PD). The signal from the photodiode is electronically demodulated via a lock‐in amplifier (LIA) after being referenced with the trigger signal from the pump source. The first harmonic of the output signal corresponds to the fundamental modulation frequency of the probe scatter induced by the pump and is referred to as the photothermal amplitude signal (PTS). Imaging is performed by raster scanning the sample stage alongside the longitudinal and transverse directions of the sample plane with a pixel dwell time of 28 ms. The sample is mounted on two stages for courser and finer resolution imaging. Specifically, a motorized stage is used for larger field of view images (larger than 20 by 20 µm), as for the data presented in Figure 2, Figures S1 and S2. For such images a 1 µm step size is used. A piezoelectric stage is mounted on the motorized stage for higher resolution imaging of smaller field of view areas (e.g., in Figure 3; Figures S5 and S6), where a 0.2 um step size is used. For simultaneous fluorescence imaging, a short‐pass dichroic mirror with a cutoff wavelength at 750 nm is placed before the probe objective, for reflection of the 980 nm probe and transmission of visible wavelengths lower than 750 nm. The LED source used for fluorescence has three channels covering altogether a spectral range from 400–700 nm. The Stokes’ shifted fluorescent emission is transmitted through the dichroic mirror and separated from the excitation via the appropriate filter cubes.

### 5.4 Image Processing

For ratio imaging between two distinct wavenumbers, normalization according to the gain curve and the incident power was performed. The power output incident on the sample used for imaging at 1660 cm^−1^ was set at 2 mW, while the power output for wavenumbers at 1625 and 1675 cm^−1^ for the same current setting were measured to be at 1.7 and 1.4 mW, respectively. Pixels that were smaller than 3 µV in value, corresponding to two times the upper limit of the system's noise (1.5 µV) were removed and set to 0. In addition, each amplitude image was smoothed by a factor of 0.5 pixels corresponding to a physical dimension of 500 nm (smaller than diffraction limited spot size).

### 5.5 Statistical Data

Statistical collection of data for the *β/α* ratio as well as *τ* values at different cell regions, was performed by freehand selection of respective areas in ImageJ from the corresponding images. The data are represented by the mean ± standard deviation, accompanied by the number of cells (N) and the total area (A) of the cell regions of interest (i.e., process or cell body), representing the number of data points in the set.

### 5.6 Spectral Acquisition and Processing

For the spectra presented, the acquisition speed per data point was set to 200 ms with a spectral resolution of 1 cm^−1^. A 2‐point Adjacent Averaging filtering was applied to the raw data for noise reduction in combination with a 1‐point upper envelope analysis to remove spectral dips associated with the presence of water vapor. Lastly, each spectrum was normalized by the appropriate gain curve collected at the same QCL current setting. For Amide I band area calculations, each individual spectrum was normalized, and the integral between 1600–1724 cm^−1^ is calculated.

### 5.7 Spectral Deconvolution

To identify individual secondary components within the Amide I band, a 10 pt window second derivative was applied to the collected spectra. Negative peaks of the second derivative within the spectral range from 1600–1700 cm^−1^ were identified and set as initial parameters in the Multiple Peak Fit tool in Origin software, assuming Lorentzian profiles. The number of iteration points was chosen to be 20,000 to ensure convergence. Each Lorentzian curve was assigned a secondary protein structure based on the following Table 1.

**TABLE 1:** Secondary protein structure peak assignment [26].

β sheets	<1636 cm^−1^ (± 2)
Random coils	1640–1648 cm^−1^ (± 2)
α‐helix	1652–1660 cm^−1^ (± 2)
β turns	1664–1670 cm^−1^ (± 2)
β sheets	>1674 cm^−1^ (± 2)

The relative contribution of each component was calculated by adding all the areas sharing the same family of protein structure and dividing it by the total area of all peaks that are between 1600 and 1700 cm^−1^.

### 5.8 Hyper‐Temporal Image Stack Acquisition and Processing

Alongside the first harmonic PTS signal, the local transient photothermal signal can be acquired, capturing the heating and diffusion dynamics within the pump period of 10 µs. For the acquisition of hyper‐temporal image stacks, a pixel dwell time of 100 ms was used to provide enough time for the storage of the entire trace per pixel. The temporal resolution of the time traces was at 9.8 ns for a demodulation frequency at 100 kHz. The 3D matrix was processed to extract images with 250 ns temporal spacing after the baseline signal (first 100 data points before the pump pulse trigger) was subtracted from each image. After subtracting the baseline value, the 1/e time decay constant was calculated by the timestamp difference between the peak signal and the first data point that was smaller in value than 1/e of the peak signal. To spatially map the 1/e time decay constant during the diffusion window, each trace was smoothed by 25 points corresponding to a 250 ns time window. For each pixel for which the mean photothermal trace over one period was equal or less than 3 µV, the time decay constant was set to 0. Statistical collection of data for *τ* values at different cell interfaces was performed by freehand selection of respective areas in ImageJ from the equivalent time decay constant images.

## Funding

M.S. acknowledges funding from the Air Force Office of Scientific Research (AFOSR) (DURIP FA9550‐18‐1‐0348) and the National Science Foundation (NSF) (ECCS‐1846659). M.S. and P.S. acknowledge funding from the Air Force Office of Scientific Research (AFOSR) (FA9550‐23‐1‐0006). GPN acknowledges NEXTGENERATIONEU (NGEU) funded by the Ministry of University and Research (MUR), National Recovery and Resilience Plan (NRRP) (project MNESYS (PE0000006)—A Multiscale integrated approach to the study of the nervous system in health and disease (DD n. 1553, 11.10.2022), project CN00000041–National Center for Gene Therapy and Drugs based on RNA Technology (DD n. 1035, 17.06.2022)) and the Air Force Office of Scientific Research (AFOSR) (FA9550‐20‐1‐0324, FA9550‐23‐1‐0583). V. B. acknowledges Air Force Office of Scientific Research (AFOSR) ASTROLIGHT (FA9550‐20‐1‐0386) and ASTROTALK (FA9550‐23‐1‐0736) and Army Research Office ASTRO‐CLUSTER W911NF2520009 (82737‐HC‐INT). G.P.N. and V.B acknowledge the Air Force Office of Scientific Research (AFOSR) AstroSense (FA9550‐24‐1‐0001). V.B. and T. P. are supported by NEXTGENERATIONEU (NGEU) funded by the Ministry of University and Research (MUR), National Recovery and Resilience Plan (NRRP), PRIN‐PNRR 2022 – NANODYN – P2022Z27NS. G. C. is supported by PNRR MUR project ECS_00000033_ECOSISTER. C.L. is supported by Air Force Office of Scientific Research (AFOSR) ASTROTALK (FA9550‐23‐1‐0736).

## Conflicts of Interest

The authors declare no conflicts of interest.

## Supporting information




**Supporting File**: advs73708‐sup‐0001‐SuppMat.docx.

## Data Availability

The data used to produce the plots within this paper are available.

## References

[1] M. Simard and M. Nedergaard , “The Neurobiology of Glia in the Context of Water and Ion Homeostasis,” Neuroscience 129 (2004): 877–896, 10.1016/j.neuroscience.2004.09.053. 15561405

[2] J. S. Jung , R. V. Bhat , G. M. Preston , W. B. Guggino , J. M. Baraban , and P. Agre , “Molecular Characterization of An Aquaporin cDNA From Brain: Candidate Osmoreceptor and Regulator of Water Balance,” Proceedings of the National Academy of Sciences 91 (1994): 13052–13056, 10.1073/pnas.91.26.13052. PMC45579 7528931

[3] J. D. Neely , M. Amiry‐Moghaddam , O. P. Ottersen , S. C. Froehner , P. Agre , and M. E. Adams , “Syntrophin‐Dependent Expression and Localization Of Aquaporin‐4 Water Channel Protein,” Proceedings of the National Academy of Sciences 98 (2001): 14108–14113, 10.1073/pnas.241508198. PMC61176 11717465

[4] O. Butenko , D. Dzamba , J. Benesova , et al., “The Increased Activity of TRPV4 Channel in the Astrocytes of the Adult Rat Hippocampus After Cerebral Hypoxia/Ischemia,” PLoS ONE 7 (2012): 39959, 10.1371/journal.pone.0039959. PMC3384594 22761937

[5] M. Santello , N. Toni , and A. Volterra , “Astrocyte Function from Information Processing to Cognition and Cognitive Impairment,” Nature Neuroscience 22 (2019): 154–166, 10.1038/s41593-018-0325-8. 30664773

[6] G. Seifert and C. Steinhäuser , “Neuron–Astrocyte Signaling and Epilepsy,” Experimental Neurology 244 (2013): 4–10, 10.1016/j.expneurol.2011.08.024. 21925173

[7] H.‐G. Lee , M. A. Wheeler , and F. J. Quintana , “Function and Therapeutic Value of Astrocytes in Neurological Diseases,” Nature Reviews Drug Discovery 21 (2022): 339–358, 10.1038/s41573-022-00390-x. 35173313 PMC9081171

[8] T. Posati , A. Pistone , E. Saracino , et al., “A Nanoscale Interface Promoting Molecular and Functional Differentiation of Neural Cells,” Scientific Reports 6 (2016): 31226, 10.1038/srep31226. 27503424 PMC4977496

[9] M. G. Mola , E. Saracino , F. Formaggio , et al., “Cell Volume Regulation Mechanisms in Differentiated Astrocytes,” Cellular Physiology and Biochemistry 55 (2021): 196–212. 34740285 10.33594/000000469

[10] K. M. O'Neill , E. Saracino , B. Barile , et al., “Decoding Natural Astrocyte Rhythms: Dynamic Actin Waves Result from Environmental Sensing by Primary Rodent Astrocytes,” Advanced Biology 7 (2023): 2200269. 10.1002/adbi.202200269 36709481

[11] A. Chari and H. Stark , “Prospects and Limitations of High‐Resolution Single‐Particle Cryo‐Electron Microscopy,” Annual Review of Biophysics 52 (2023): 391–411, 10.1146/annurev-biophys-111622-091300. 37159297

[12] P. Anantha , P. Raj , E. Saracino , et al., “Uncovering Astrocyte Morphological Dynamics Using Optical Diffraction Tomography and Shape‐Based Trajectory Inference,” Advanced Healthcare Materials 14 (2025): 2402960, 10.1002/adhm.202402960. 39740118

[13] Q. Xia , J. Yin , Z. Guo , and J.‐X. Cheng , “Mid‐Infrared Photothermal Microscopy: Principle, Instrumentation, and Applications,” The Journal of Physical Chemistry B 126 (2022): 8597–8613, 10.1021/acs.jpcb.2c05827. 36285985

[14] P. D. Samolis , X. Zhu , and M. Y. Sander , “Time‐Resolved Mid‐Infrared Photothermal Microscopy for Imaging Water‐Embedded Axon Bundles,” Analytical Chemistry 95 (2023): 16514–16521, 10.1021/acs.analchem.3c02352. 37880191 PMC10652238

[15] Y. Bai , D. Zhang , L. Lan , et al., “Ultrafast Chemical Imaging by Widefield Photothermal Sensing Of Infrared Absorption,” Science Advances 5 (2019): aav7127, 10.1126/sciadv.aav7127. PMC6641941 31334347

[16] E. M. Paiva and F. M. Schmidt , “Ultrafast Widefield Mid‐Infrared Photothermal Heterodyne Imaging,” Analytical Chemistry 94 (2022): 14242–14250, 10.1021/acs.analchem.2c02548. 36197677 PMC9583073

[17] K. Kniazev , E. Zaitsev , S. Zhang , et al., “Hyperspectral and Nanosecond Temporal Resolution Widefield Infrared Photothermal Heterodyne Imaging,” ACS Photonics 2023, 10, 2854–2860.

[18] J. Zhao , L. Jiang , A. Matlock , et al., “Mid‐Infrared Chemical Imaging of Intracellular Tau Fibrils Using Fluorescence‐Guided Computational Photothermal Microscopy,” Light: Science & Applications 12 (2023): 147, 10.1038/s41377-023-01191-6. PMC10272128 37322011

[19] P. Fu , W. Cao , T. Chen , et al., “Super‐Resolution Imaging of Non‐Fluorescent Molecules by Photothermal Relaxation Localization Microscopy,” Nature Photonics 17 (2023): 330–337, 10.1038/s41566-022-01143-3.

[20] J. Yin , M. Zhang , Y. Tan , et al., “Video‐Rate Mid‐Infrared Photothermal Imaging by Single‐Pulse Photothermal Detection Per Pixel,” Science Advances 9 (2023): adg8814, 10.1126/sciadv.adg8814. PMC10266719 37315131

[21] G. Ishigane , K. Toda , M. Tamamitsu , H. Shimada , V. R. Badarla , and T. Ideguchi , “Label‐Free Mid‐Infrared Photothermal Live‐Cell Imaging Beyond Video Rate,” Light: Science & Applications 12 (2023): 174, 10.1038/s41377-023-01214-2. PMC10354118 37463888

[22] P. D. Samolis and M. Y. Sander , “Increasing Contrast in Water‐Embedded Particles via Time‐Gated Mid‐Infrared Photothermal Microscopy,” Optics Letters 49 (2024): 1457–1460, 10.1364/OL.513742. 38489424

[23] Y. Zhang , H. Zong , C. Zong , et al., “Fluorescence‐Detected Mid‐Infrared Photothermal Microscopy,” Journal of the American Chemical Society 143 (2021): 11490–11499, 10.1021/jacs.1c03642. 34264654 PMC8750559

[24] Y. Zhu , X. Ge , H. Ni , et al., “Stimulated Raman Photothermal Microscopy Towards Ultrasensitive Chemical Imaging,” Science Advances 9 (2023): eadi2181, 10.1126/sciadv.adi2181. 37889965 PMC10610916

[25] M. Tamamitsu , K. Toda , H. Shimada , et al., “Label‐Free Biochemical Quantitative Phase Imaging With Mid‐Infrared Photothermal Effect,” Optica 7 (2020): 359–366, 10.1364/OPTICA.390186.

[26] A. Barth , “Infrared Spectroscopy of Proteins,” Biochimica et Biophysica Acta (BBA)—Bioenergetics 1767 (2007): 1073–1101, 10.1016/j.bbabio.2007.06.004. 17692815

[27] P. D. Samolis , D. Langley , B. M. O'Reilly , et al., “Label‐Free Imaging of Fibroblast Membrane Interfaces and Protein Signatures With Vibrational Infrared Photothermal and Phase Signals,” Biomedical Optics Express 12 (2021): 303–319, 10.1364/BOE.411888. 33520386 PMC7818956

[28] N. Gustavsson , A. Paulus , I. Martinsson , et al., “Correlative Optical Photothermal Infrared and X‐Ray Fluorescence for Chemical Imaging of Trace Elements and Relevant Molecular Structures Directly in Neurons,” Light: Science & Applications 10 (2021): 151, 10.1038/s41377-021-00590-x. PMC8298485 34294676

[29] J. M. Lim , C. Park , J.‐S. Park , C. Kim , B. Chon , and M. Cho , “Cytoplasmic Protein Imaging With Mid‐Infrared Photothermal Microscopy: Cellular Dynamics of Live Neurons and Oligodendrocytes,” The Journal of Physical Chemistry Letters 10 (2019): 2857–2861, 10.1021/acs.jpclett.9b00616. 31025568

[30] R. Bolarinho , J. Yin , H. Ni , Q. Xia , and J.‐X. Cheng , “Background‐Free Mid‐Infrared Photothermal Microscopy via Single‐Shot Measurement of Thermal Decay,” Analytical Chemistry 97 (2025): 4299–4307, 10.1021/acs.analchem.4c03689. 39965086 PMC12236061

[31] J. Yin , L. Lan , Y. Zhang , et al., “Nanosecond‐Resolution Photothermal Dynamic Imaging via MHZ Digitization and Match Filtering,” Nature Communications 12 (2021): 7097, 10.1038/s41467-021-27362-w. PMC8651735 34876556

[32] X. Teng , M. Li , H. He , et al., “Mid‐Infrared Photothermal Imaging: Instrument and Life Science Applications,” Analytical Chemistry 96 (2024): 7895–7906, 10.1021/acs.analchem.4c02017. 38702858 PMC11785416

[33] V. Benfenati and S. Ferroni , in Homeostatic Control of Brain Function, eds. D. Boison and S. A. Masino (Oxford University Press 2015).

[34] Z. Zhou , J. Zhan , Q. Cai , et al., “The Water Transport System in Astrocytes–Aquaporins,” Cells 11 (2022): 2564, 10.3390/cells11162564. 36010640 PMC9406552

[35] A. S. Verkman and A. K. Mitra , “Structure and Function of Aquaporin Water Channels,” American Journal of Physiology‐Renal Physiology 278 (2000): F13–F28, 10.1152/ajprenal.2000.278.1.F13. 10644652

[36] A. S. Verkman , P. Phuan , N. Asavapanumas , and L. Tradtrantip , “Biology of AQP4 and Anti‐ AQP4 Antibody: Therapeutic Implications for NMO,” Brain Pathology 23 (2013): 684–695, 10.1111/bpa.12085. 24118484 PMC3890327

[37] A. S. Verkman , J. Ratelade , A. Rossi , H. Zhang , and L. Tradtrantip , “Aquaporin‐4: Orthogonal Array Assembly, CNS Functions, and Role in Neuromyelitis Optica,” Acta Pharmacologica Sinica 32 (2011): 702–710, 10.1038/aps.2011.27. 21552296 PMC3601948

[38] J. Szu , V. Parpura , M. Amiry‐Moghaddam , and A. J. Smith , “Editorial: Mechanisms and Consequences of Aquaporin‐4 Redistribution in Neurological Disease,” Frontiers in Cellular Neuroscience 17 (2023): 1143352, 10.3389/fncel.2023.1143352. 36816853 PMC9930891

[39] J. Jorgačevski , R. Zorec , and M. Potokar , “Insights Into Cell Surface Expression, Supramolecular Organization, and Functions of Aquaporin 4 Isoforms in Astrocytes,” Cells 9 (2020): 2622, 10.3390/cells9122622. 33297299 PMC7762321

[40] F. Lang , G. L. Busch , M. Ritter , et al., “Functional Significance of Cell Volume Regulatory Mechanisms,” Physiological Reviews 78 (1998): 247–306, 10.1152/physrev.1998.78.1.247. 9457175

[41] V. Benfenati , M. Amiry‐Moghaddam , M. Caprini , et al., “Expression and Functional Characterization of Transient Receptor Potential Vanilloid‐Related Channel 4 (TRPV4) in Rat Cortical Astrocytes,” Neuroscience 148 (2007): 876–892, 10.1016/j.neuroscience.2007.06.039. 17719182

[42] R. Sánchez‐Hernández , M. Benítez‐Angeles , A. M. Hernández‐Vega , and T. Rosenbaum , “Recent Advances on the Structure and the Function Relationships of the TRPV4 Ion Channel,” Channels 18: 2313323, 10.1080/19336950.2024.2313323. PMC10868539 38354101

[43] V. Benfenati , G. P. Nicchia , M. Svelto , C. Rapisarda , A. Frigeri , and S. Ferroni , “Functional Down‐Regulation of Volume‐Regulated Anion Channels in AQP4 Knockdown Cultured Rat Cortical Astrocytes,” Journal of Neurochemistry 100 (2007): 87–104, 10.1111/j.1471-4159.2006.04164.x. 17064359

[44] M. Amiry‐Moghaddam and O. P. Ottersen , “The Molecular Basis of Water Transport in the Brain,” Nature Reviews Neuroscience 4 (2003): 991–1001, 10.1038/nrn1252. 14682361

[45] A. Messing and M. Brenner , “GFAP at 50,” ASN Neuro 12 (2020): 1759091420949680, 10.1177/1759091420949680. 32811163 PMC7440737

[46] C.‐A. Schoenenberger , H. G. Mannherz , and B. M. Jockusch , “Actin: From Structural Plasticity to Functional Diversity,” European Journal of Cell Biology 90 (2011): 797–804, 10.1016/j.ejcb.2011.05.002. 21820202

[47] X. Chen , S. J. Roeters , F. Cavanna , J. Alvarado , and C. R. Baiz , “Crowding Alters F‐Actin Secondary Structure and Hydration,” Communications Biology 6 (2023): 900. 37660224 10.1038/s42003-023-05274-3 PMC10475093

[48] J. Joseph , L. Spantzel , M. Ali , et al., “Nanoscale Chemical Characterization of Secondary Protein Structure of F‐Actin Using Mid‐Infrared Photoinduced Force Microscopy (PiF‐IR),” Spectrochimica Acta Part A: Molecular and Biomolecular Spectroscopy 306 (2024): 123612, 10.1016/j.saa.2023.123612. 37931494

[49] G. P. Nicchia , M. Srinivas , W. Li , C. F. Brosnan , A. Frigeri , and D. C. Spray , “New Possible Roles for Aquaporin‐4 in Astrocytes: Cell Cytoskeleton and Functional Relationship With Connexin43,” The FASEB Journal 19 (2005): 1674–1676, 10.1096/fj.04-3281fje. 16103109

[50] E. Guadagno and H. Moukhles , “Laminin‐Induced Aggregation of The Inwardly Rectifying Potassium Channel, Kir4.1, and the Water‐Permeable Channel, AQP4, via A Dystroglycan‐Containing Complex In Astrocytes,” Glia 47 (2004): 138–149, 10.1002/glia.20039. 15185393

[51] L. Dobson , I. Reményi , and G. E. Tusnády , “The human Transmembrane Proteome,” Biology Direct 10 (2015): 31, 10.1186/s13062-015-0061-x. 26018427 PMC4445273

[52] M. S. Almén , K. J. V. Nordström , R. Fredriksson , and H. B. Schiöth , “Mapping the Human Membrane Proteome: A Majority of the Human Membrane Proteins can be Classified According to Function and Evolutionary Origin,” BMC Biology 7 (2009): 50. 19678920 10.1186/1741-7007-7-50 PMC2739160

[53] J. S. Soto , Y. Jami‐Alahmadi , J. Chacon , et al., “Astrocyte–Neuron Subproteomes and Obsessive–Compulsive Disorder Mechanisms,” Nature 616 (2023): 764–773, 10.1038/s41586-023-05927-7. 37046092 PMC10132990

[54] T. Kawaguchi , H. Honda , K. Hatori , E. Imai , and K. Matsuno , “Fourier's Law of Heat Transfer and Its Implication to Cell Motility,” Bio Systems 81 (2005): 19–24, 10.1016/j.biosystems.2005.01.003. 15917124

[55] D. M. Leitner , “Energy Flow in Proteins,” Annual Review of Physical Chemistry 59 (2008): 233–259, 10.1146/annurev.physchem.59.032607.093606. 18393676

[56] A. Xie , L. van der Meer , W. Hoff , and R. H. Austin , “Long‐Lived Amide I Vibrational Modes in Myoglobin,” Physical Review Letters 84 (2000): 5435, 10.1103/PhysRevLett.84.5435. 10990962

[57] X. Yu and D. M. Leitner , “Heat Flow in Proteins: Computation of Thermal Transport Coefficients,” The Journal of Chemical Physics 122 (2005): 054902, 10.1063/1.1830431. 15740348

[58] L. K. Tamm , H. Hong , and B. Liang , “Folding and Assembly of β‐Barrel Membrane Proteins,” Biochimica et Biophysica Acta (BBA)—Biomembranes 1666 (2004): 250–263, 10.1016/j.bbamem.2004.06.011. 15519319

[59] A. Lervik , F. Bresme , S. Kjelstrup , D. Bedeaux , and J. M. Rubi , "Heat transfer in protein–water interfaces," Physical Chemistry Chemical Physics 12 (2010): 1610–1617, 10.1039/B918607G. 20126777

[60] M. Amiry‐Moghaddam , R. Xue , F.‐M. Haug , et al., “Alpha Syntrophin Deletion Removes The Perivascular But Not The Endothelial Pool Of Aquaporin‐4 at the Blood‐Brain Barrier and Delays the Development of Brain Edema in An Experimental Model of Acute Hyponatremia,” The FASEB Journal 18 (2004): 542–544, 10.1096/fj.03-0869fje. 14734638

[61] L. K. Lunde , L. M. A. Camassa , E. H. Hoddevik , et al., “Postnatal Development of the Molecular Complex Underlying Astrocyte Polarization,” Brain Structure and Function 220 (2015): 2087–2101, 10.1007/s00429-014-0775-z. 24777283 PMC4481305

[62] A. Cibelli , M. G. Mola , E. Saracino , et al., “Aquaporin‐4 and Transient Receptor Potential Vanilloid 4 Balance in Early Postnatal Neurodevelopment,” Glia 72 (2024): 938–959, 10.1002/glia.24512. 38362923

[63] M. Lattke and F. Guillemot , “Understanding Astrocyte Differentiation: Clinical Relevance, Technical Challenges, and New Opportunities in The Omics Era,” WIREs Mechanisms of Disease 14 (2022): 1557, 10.1002/wsbm.1557. PMC9539907 35546493

[64] C. Escartin , E. Galea , A. Lakatos , et al., “Reactive Astrocyte Nomenclature, Definitions, and Future Directions,” Nature Neuroscience 24 (2021): 312–325, 10.1038/s41593-020-00783-4. 33589835 PMC8007081

[65] A. Volterra and J. Meldolesi , “Astrocytes, From Brain Glue to Communication Elements: The Revolution Continues,” Nature Reviews Neuroscience 6 (2005): 626–640, 10.1038/nrn1722. 16025096

