# Supplementary material for: Label‐Free Molecular Characterization of Protein Aggregates in Differentiated Astrocytes
Source: Adv Sci (Weinh). 2026 Jan 7;13(16):e15228. doi: 10.1002/advs.202515228 (PMC13042664; doi:10.1002/advs.202515228)
Supplement: Supplementary file 1 — Supporting File: advs73708‐sup‐0001‐SuppMat.docx. [file ADVS-13-e15228-s001.docx]

Supporting Information

Label-free molecular characterization of protein aggregates in differentiated astrocytes

Panagis D. Samolis^1,2^, Chiara Lazzarini^3,4^, Rahmetullah Durgun^1,2^, Barbara Barile^4^, Giorgia Conte^3^, Tamara Posati^3^, Marco Caprini^5^, Grazia Paola Nicchia^4^, Valentina Benfenati^3^, Michelle Y. Sander^1,2,6,7^*

For the HTlc cells (H1-H4) presented in Figure S1, four additional cells were analyzed and their characteristic spectra at the astrocyte processes and cell body compared. As shown in the main manuscript, the soma features low frequency broadening and thus a higher signal at spectral regions around 1625 cm^-1^ associated with β-sheets. The displayed β/α ratio values correspond to photothermal measurements at 1625 cm^-1^ over 1660 cm^-1^.





**Figure. S1.**  **Secondary protein structure in differentiated astrocytes.** Four differentiated primary rat astrocyte cells on a HTlc substrate (H1-H4) with extensions from cell body in starlike morphology (A) Photothermal imaging at the Amide I protein band at 1660 cm^-1^. (B) Ratio imaging of photothermal signal at 1625 cm^-1^ (peak for β-sheet proteins) over 1660 cm^-1^ (peak for α-helix proteins) highlighting variations of secondary protein conformation. HTlc cell processes from cells H1, H2, H3 and H4 are characterized by a ratio (β/α) ~ 0.72, ~ 0.71, ~ 0.77, ~ 0.7, respectively. The cell main body in all cells H1, H2, H3 and H4 contain regions with higher content of β-sheet proteins with the ratio of (β/α) ~ 0.88, ~ 1, ~ 0.9, ~ 1.3, respectively. (C) Selected characteristic spectra at the HTlc processes (shown in solid blue- from the location denoted by the blue or white arrows in Figure S1 b) and at clusters of β-sheets (dashed dotted dark yellow-for location, see yellow arrow in Figure S1 b). (D) Deconvolved fitted spectra (dashed red) demonstrating individual secondary protein structure components for the astrocyte processes, with α-helix content ranges from 19-58% (cyan) and β-sheet content from 34-50 % (yellow for low wavenumber β-sheet, orange for high wavenumber β-sheet).

For the PDL cells (P1-P5) presented in Figure S2, deviations from the characteristic spectra obtained at the cell processes of HTlc cells, include high frequency spectral broadening and thus higher signal at spectral regions around 1675 cm^-1^ associated also with β sheets. Thus, the β/α ratio values correspond to a photothermal signal at 1675 cm^-1^ over 1660 cm^-1^.

**

**

**Figure S2.**  **Secondary protein structure in non-differentiated astrocytes.** Non-differentiated primary rat astrocyte cell on a PDL substrate with polygonal shape. (A) Photothermal imaging at the Amide I protein band at 1660 cm^-1^. (B) Ratio imaging of photothermal signal at 1675 cm^-1^ (peak for β-sheet proteins) over 1660 cm^-1^ (peak for α-helix proteins), highlighting the homogenous distribution of secondary protein conformation characterized by a ratio of (β/α) ~ 0.9-1, indicating high content of β-sheet proteins. (C) Characteristic broad Amide I spectra at cell body (shown in dark yellow). (D) Deconvolved fitted spectra (dashed red) demonstrating individual secondary protein structure components, with α-helical content ranging from 2-28% (cyan) and β-sheet content from 70-98 % (yellow for low wavenumber β-sheets, orange for high wavenumber β-sheets).

In Figure S3, the distribution of β/α values per cell are presented in a box chart graph. The data is obtained from 21 processes collected from 6 HTlc cells (see blue dots – data labeled with p), the cell body of 6 HTlc cells (see orange dots – data labeled with b) as well as from the cell body of 6 PDL cells (see dark yellow dots – data labeled with b). Statistical analysis is performed for each data set (see Methods). The box boundaries correspond to the 16% and 84% percentile values, containing the data points within one standard deviation of the mean. A clear trend is visible in the β*/*α ratio for the different cell types: For the HTlc astrocytes, the processes are characterized by a mean β/α ratio of 0.78 ± 0.09 (number of cells N=6, associated area for evaluation A=11,606 µm^2^), while the β/α values at the cell body have a mean of 0.8 ± 0.09 (N=6, A=7,479 µm^2^). Although β-sheet clusters in the cell body of HTlc cells can be clearly identified, the localized nature of the β-sheet clusters forms overall a small fraction of the data points small fraction of the data points. This is not sufficient to result in a statistically significant increase of the mean, since this measure does not account for the high spatial density of such signatures.

For the PDL undifferentiated astrocytes, a 1.3-fold increase in the mean β/α value compared to the cell processes of HTlc cells, at 0.93± 0.07 (N=6, A=25,949 µm^2^) is observed. This distribution provides a statistically significant result with a p-value of 0.0003 when comparing the mean ratio of HTlc cell processes to the PDL cell bodies and a p-value of 0.0005 when comparing the mean ratio of HTlc cell bodies to the PDL cell bodies.


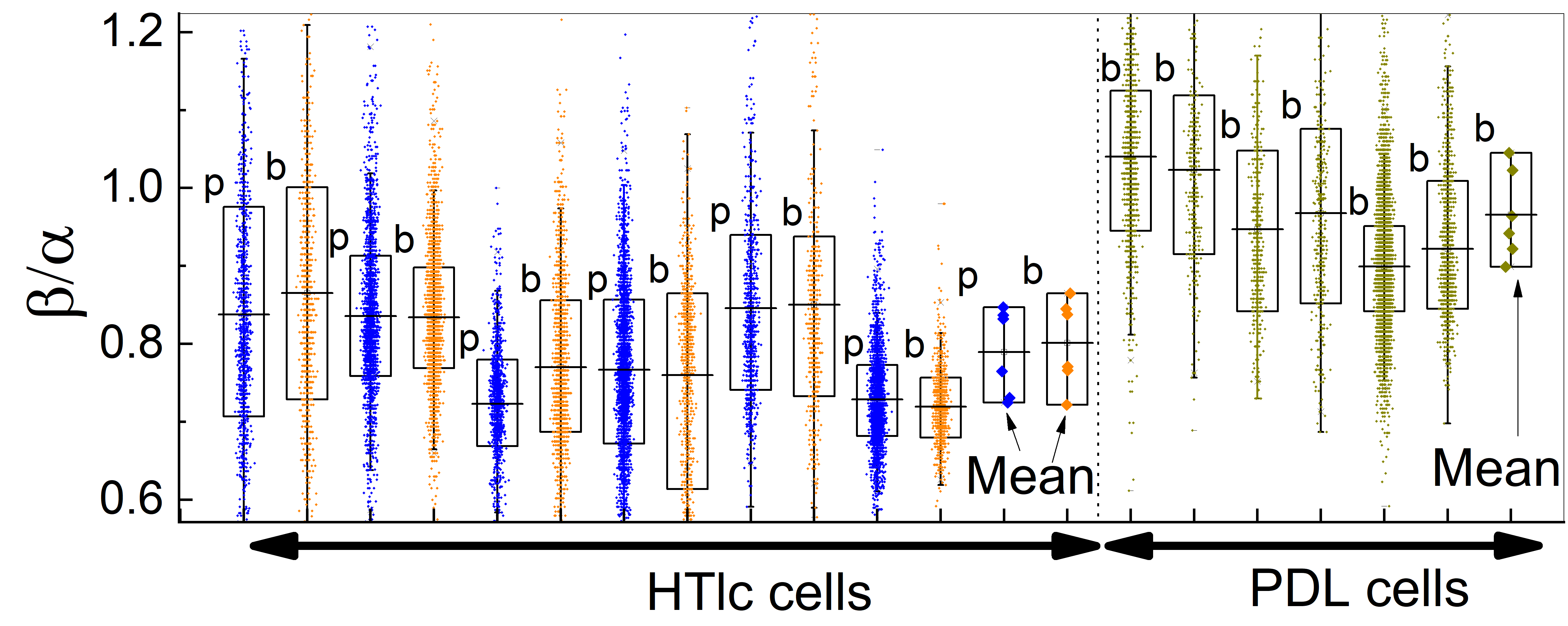


**Figure S3.**  **Statistical distribution of β/α ratio values per cell**. Box chart graph of β/α values per cell measured from 21 HTlc cell processes from 6 cells (see blue dots – data labelled with p), the cell body of 6 HTlc cells (see orange dots – data labelled with b) of differentiated astrocyte cells where each blue-orange neighbouring pair originate from the same cell. In addition, the β/α ratio values from the cell body of 6 non-differentiated PDL cells (see dark yellow – data labelled with b) are presented. The mean β/α ratio of each cell distribution are also plotted in a similar fashion. The mean value for the HTlc cell processes is found to be 0.78 ± 0.09 (N=6, A=11,606 µm^2^), and at the cell body at 0.8 ± 0.09 (N=6, A=7,479 µm^2^). A larger mean at 0.93 ± 0.07 (N=6, A=25,949 µm^2^) is found for the PDL cells.

**Figure S4. Box chart graphs of random coil content.** (A) Random coil content is found to be consistently less than 3% in PDL cytoplasm, while its presence in 3 HTlc astrocyte processes gives a mean of around 12%. (B) The cumulative content of α-helix and random coil, has a mean of 50% ±7% for the HTlc processes.


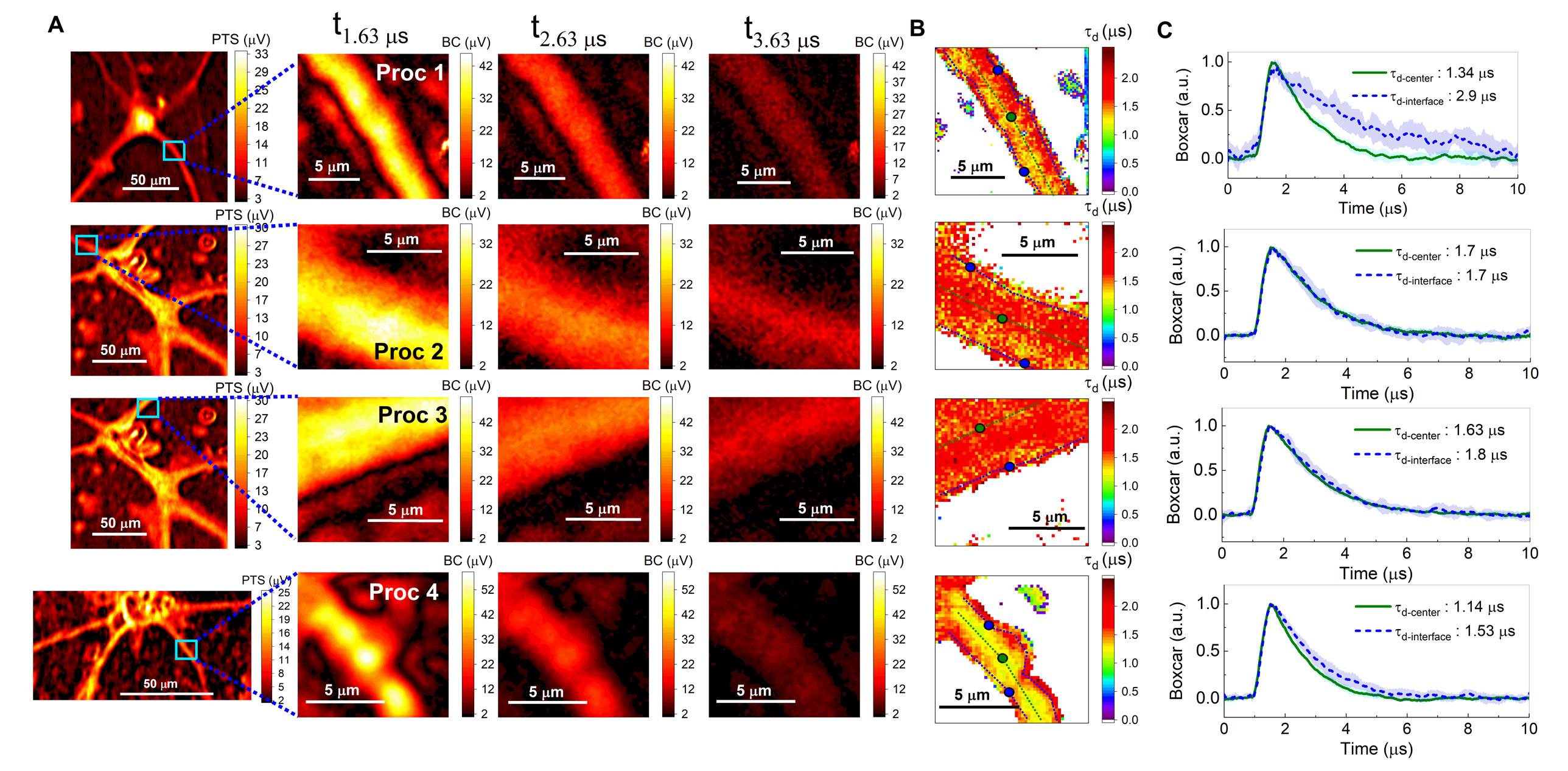


**Figure S5.**  **Thermal diffusion dynamics of astrocyte processes.** (A) Boxcar photothermal images of 4 cytoplasmic processes (Proc 1-4) from three differentiated astrocyte cells grown on HTlc at selected points in time during the diffusion window with 1 μs time separation including at t=1.63 μs, t=2.63 μs and t=3.63 μs. (B) A two-dimensional map of the 1/e time decay constant at the HTlc cell processes. (C) The mean transient curves at the interfaces of Proc 1 and Proc 4 (see dashed lines across the blue dots) show clearly slower decay rates than the mean transient curves at cell process center (see dashed lines across green dots) with τ_d-interface_=2.9 μs > τ_d-center_=1.34 μs and τ_d-interface_=1.53 μs > τ_d-center_=1. 14 μs for Proc 1 and Proc 4 respectively. Processes 2 and 3 (which are part of the same HTlc cell), show similar decay rates at the center with τ_d-center_ ~ 1.7 μs and interface τ_d-interface_ ~ 1.8 μs.


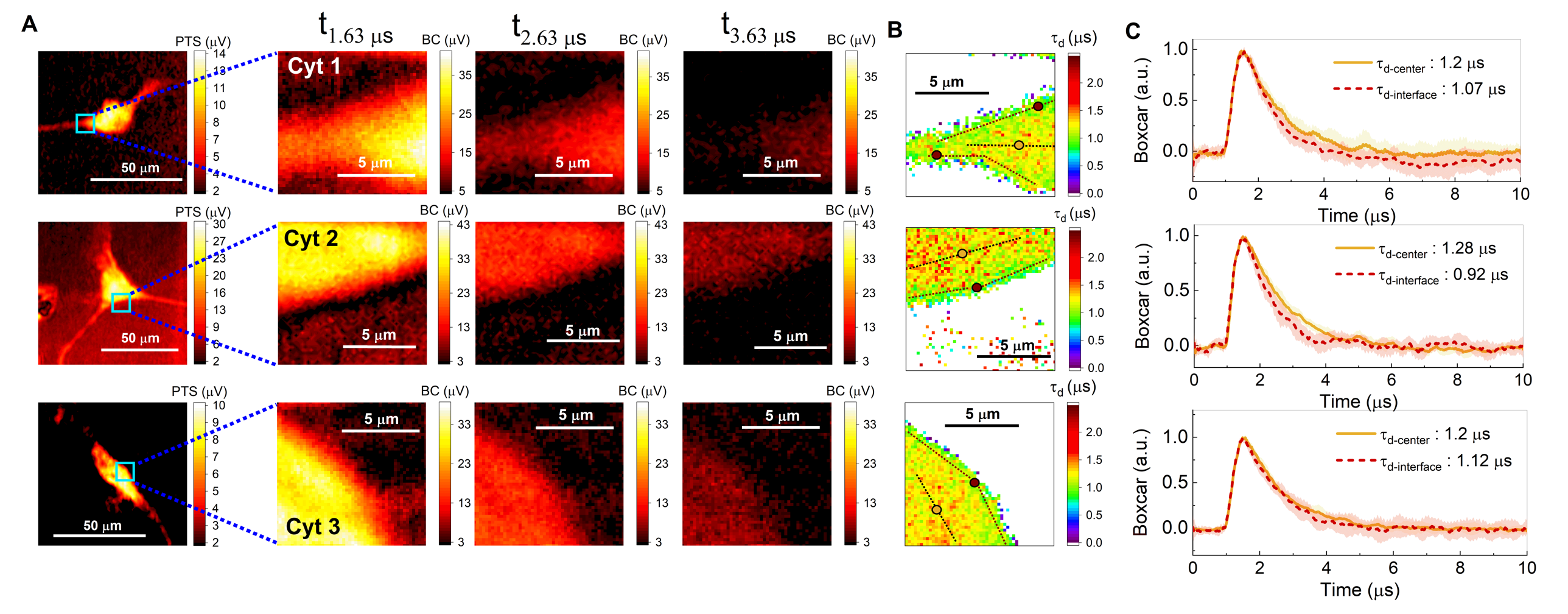


Figure S6. Thermal diffusion dynamics of cytoplasm from non-differentiated cells. (A) Photothermal amplitude images (first column) and zoom-in boxcar photothermal images of three cytoplasm interfaces (Cyt 1-3) from non-differentiated astrocyte cells grown on PDL during the diffusion window at t=1.63 μs, t=2.63 μs and t=3.63 μs. (B) A two-dimensional map of the 1/e time decay constant at the PDL cell cytoplasm. (C) The mean transient curves measured at the high signal cytoplasm locations (see dashed lines across yellow dots) have a slightly larger time decay constant τ_d_ than the constants measured at the cytoplasm interface (see dashed lines across brown dots) with τ_d-center_=1.2-1.28 μs > τ_d-interface_=0.92-1.12 μs, showing a consistently faster diffusion time at the cell interface for non-differentiated cells.
